# Force Distribution of a Novel Core-Reinforced Multilayered Mandibular Advancement Device

**DOI:** 10.3390/s21103383

**Published:** 2021-05-12

**Authors:** Hyo-Won Ahn, Soo-Yeon Lee, Hobeen Yu, Jin-Young Park, Kyung-A Kim, Su-Jung Kim

**Affiliations:** 1 Department of Orthodontics, Kyung Hee University School of Dentistry, Seoul 02447, Korea; hyowon@khu.ac.kr (H.-W.A.); k2aortho@khu.ac.kr (K.-A.K.); 2 Department of Dentistry, Graduate School, Kyung Hee University, Seoul 02447, Korea; syoun8401@naver.com (S.-Y.L.); beeny@khu.ac.kr (H.Y.); cmvjall@naver.com (J.-Y.P.)

**Keywords:** mandibular advancement devices, core-reinforced, multilayer, force distribution, dental side effects

## Abstract

A mandibular advancement device (MAD) is a commonly used treatment modality for patients with mild-to-moderate obstructive sleep apnea. Although MADs have excellent therapeutic efficacy, dental side effects were observed with long-term use of MADs. The aim of this study was to analyze the force distribution on the entire dentition according to the materials and design of the MADs. Three types of MADs were applied: model 1 (single layer of polyethylene terephthalate glycol (PETG)), model 2 (double layer of PETG + thermoplastic polyurethane (TPU)), and model 3 (core-reinforced multilayer). In the maxilla, regardless of the model, the incisors showed the lowest force distribution. In most tooth positions, the force distribution was lower in models 2 and 3 than in model 1. In the mandible, the mandibular second molar showed a significantly lower force in all models. The mandibular incisors, canines, and molars showed the highest force values in model 1 and the lowest values in model 3. Depending on the material and design of the device, the biomechanical effect on the dentition varies, and the core-reinforced multilayered MAD can reduce the force delivered to the dentition more effectively than the conventional single- or double-layer devices.

## 1. Introduction

Obstructive sleep apnea (OSA) is a common sleep disorder characterized by repeated obstruction of the upper airway during sleep, and it is highly prevalent in the elderly population [1]. Continuous positive airway pressure (CPAP) is the first-line treatment for adult patients with moderate-to-severe OSA. Despite its benefits, a significant proportion of patients are unable to tolerate CPAP [2]. Currently, mandibular advancement devices (MADs) are widely used to treat OSA patients with mild-to-moderate symptoms who are unable to tolerate CPAP therapy [3,4,5,6]. According to the clinical guidelines of the American Academy of Sleep Medicine (AASM) in 2015 [7], the improvement in quality of life by customized and titratable MADs is not inferior to that reported with CPAP therapy. Similarly, long-term study showed that both CPAP and MAD therapy demonstrated good and stable treatment effects over a 10-year follow-up [8]. As the therapeutic value of MADs became important, the role of dentists in dealing with MADs is being increasingly emphasized. A qualified dentist should have the skill to choose the appropriate MAD and make necessary modifications to accommodate patients.

The working principle of MADs is fundamentally similar to that of the functional appliances used for the correction of skeletal Class II discrepancies [4]. The MAD leads to protrusion of the mandible, which increases the upper airway caliber, especially regarding the volume of the velopharynx. They are anchored mainly onto the dentition rather than the mucosa for retention [9,10]; the mandible moves into a protrusive position as clinically determined, and the device generates reciprocal forces on the dentition and the mandible [11,12,13,14,15,16].

The working mechanism of MADs inevitably results in several side effects compared with CPAP use. Short-term side effects of MADs include temporomandibular joint (TMJ) pain, discomfort in the mandibular musculature, tooth pain, excessive salivation, dry mouth, gum irritation, and a sensation of altered dental occlusion [11,12,13,14,15,16]. These mild and transient effects decrease with time and most patients can tolerate them [17]. In contrast, the long-term side effects include TMJ repositioning, or irreversible occlusal changes such as a reduction in overbite and overjet, mesial shift of the mandibular molars, and a decrease in the occlusal contact area (OCA) around the molar and premolar regions [10,12,18,19,20]. MADs induce anterior repositioning of TMJ [20], and as the mandible attempts to return to its original position during muscle relaxation, the labially directed forces are transmitted to the lower incisors and the lingually directed forces are transmitted to the upper incisors [9,17]. Ueda et al. [18] reported that for 45 OSA patients treated with MADs, a significant change in total OCA was identified in 87% of patients. They showed a tendency for a decrease in OCA on the first molar area, whereas there was an increase on the second molar area. The authors’ explanation for these changes in the OCA was mesial tipping of the second molar.

Few studies attempted to determine the predictors of long-term dental side effects because of the prolonged use of MADs [3,10,21]. To minimize the adverse effects of the device and increase patient compliance, MADs with various configurations and materials were introduced. Fritsch et al. [12] found no differences in the orthodontic side effects between the Herbst two-piece appliances and the hard acrylic mono-block type. Marklund et al. [21] compared the dental side effects of elastomeric devices with those of hard acrylic devices. They reported that soft elastomeric devices reduced dental side effects during the treatment of sleep apnea. Ahn et al. [22] designed a multilayered hybrid device, which included an outer layer of polyethylene terephthalate glycol (PETG), a middle layer of thermoplastic polyurethane (TPU), and an inner layer of the reinforced resin core. This multilayer device had enhanced durability and fracture resistance.

An analysis of the force system applied to the dentition should precede the anticipation of the direction and extent of tooth movement after the use of MADs. Recently, Lee et al. [23] analyzed the biomechanical effect of different protrusion positions of a MAD on the teeth and facial bones using finite element analysis (FEM). According to their results, both the incisors and mandibular molars, especially the buccal surface of second molars, were subjected to high stress, which caused lingual inclination of the molars.

So far, there were few studies on whether there is a difference in force distribution according to the individual tooth position or depending on the materials and design of the MADs. The aim of this study was to analyze the biomechanical effects of a new type of core-reinforced multilayered MAD on the maxillary and mandibular dentition. The hypothesis of our study is that core-reinforced multilayered MADs lower the forces delivered to the dentition better than the single- or double-layer MADs.

## 2. Materials and Methods

### 2.1. Study Design & Protocol

**Figure 1:**
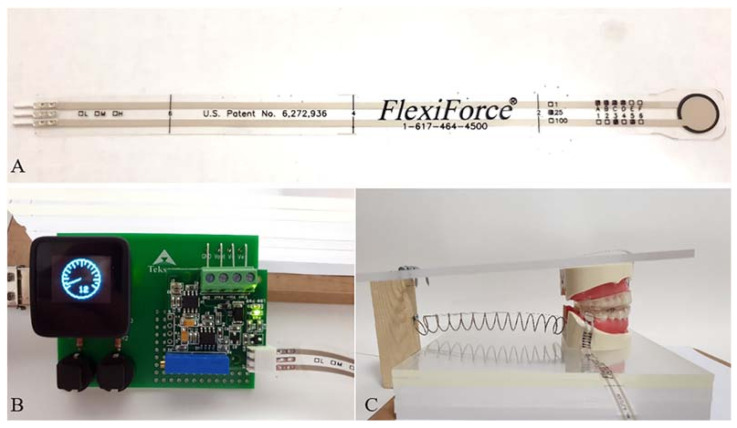
(**A**) Force sensor (FlexiForce sensor A-201, Tekscan, Boston, MA, USA) used in this study. (**B**) A development board showing measured force. (**C**) Activated state by applying MAD.

This in vitro study was designed using a dentiform (Nissin dental model, D16DP-500A.MF, Japan) and artificial teeth. The maxillary dentiform was fixed on an acrylic plate, and the mandibular dentiform was protruded to a prefixed position with three different MADs (as illustrated in Figure 1). The posterior restorative force produced from the relaxation of the mandibular muscles was represented by the restoring force of the spring. The posterior restorative force on the mandible is primarily caused by stretching of the two masticatory muscles, the deep masseter and posterior temporal muscle [23]. The spring tension stiffness values of these two muscles were 16.35 N/mm and 13 N/mm, respectively [24], and the force values generated by the MADs were approximately 100 gF for each millimeter of advancement [25]. According to Cohen–Levy’s in vivo study [26], the force generated by MADs was 1.18 N/mm. Considering that the general mandibular advancement is 4–6 mm [9,19,21], we set the restorative force of the spring to 700 gF in this study.

### 2.2. Force Sensor

The FlexiForce sensor (FlexiForce sensor A-201, Tekscan, Boston, MA, USA) was used, which is very thin (0.2 mm thick), flexible, and easy to manipulate (as illustrated in Figure 1). The sensor diameter is 9.53 mm and can be adjusted by cutting out the margin with scissors. The measurable force ranged from 0–25 pounds (110 N). All the force values were measured using a single sensor. The value measured from the sensor was digitalized by a development board and reported by an arbitrary unit (as illustrated in Figure 1). The sensor was attached to the tooth surface using a double-sided tape (ScotchTM, 3M), as recommended by the manufacturer.

The force delivered to the upper and lower dentition with three different MADs was measured 30 times. To minimize the measurement error, the force was measured separately for each target tooth. The subject tooth surfaces were cut uniformly to the thickness of the sensor (0.2 mm). The force sensed by the sensor was calibrated, and no adjustments were made before the spring was activated.

### 2.3. Fabrication of the Three Types of Mandibular Advancement Devices (MADs)

**Figure 2:**
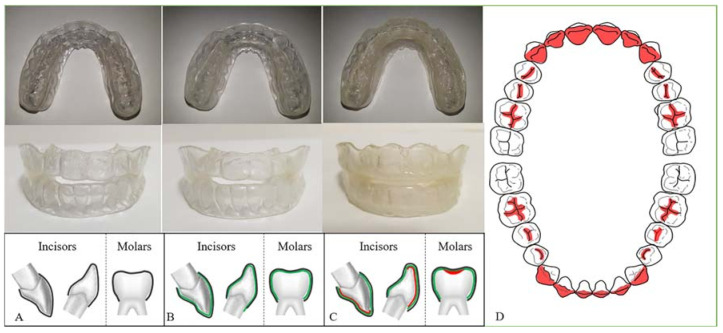
Three types of one-piece mandibular advancement devices (MADs) used in this study. They were made of different materials and compositions; however, the overall configuration and thickness of the device was controlled. (**A**) Model 1, a single-layer MAD (thickness 4 mm, PETG); (**B**) model 2, a double-layer MAD (thickness 4 mm, outer: PETG/ inner: TPU); (**C**) model 3, a triple-layer MAD (thickness 4–4.5 mm, inner: reinforced resin core/ middle: TPU/ outer: PETG); (**D**) resin core area in model 3. PETG, polyethylene terephthalate glycol; TPU, thermoplastic polyurethane; Mx, maxilla; Mn, mandible.

In this study, one-piece MADs were used to minimize the error caused by the intra-device hinge movement of two-piece MADs. Three types of MADs, named models 1, 2, and 3, were used according to the material and design (as illustrated in Figure 2). All MADs were fabricated under the same construction bite, with 6 mm protrusion and a vertical opening of 4 mm at the molar and 6 mm at the incisor region. Model 1 was a single-layer device made of a hard thermoplastic polymer (polyethylene terephthalate glycol (PETG)) with a thickness of 2 mm. Model 2 comprised a double layer: an inner soft thermoplastic polymer (thermoplastic polyurethane (TPU), 1 mm) surrounded by PETG (1 mm). Model 3 had a triple layer, including an inner most reinforced resin core surrounded by a soft TPU and a hard PETG layer, which constituted the outermost layer. The resin core was selectively located at the incisal edge and lingual surface of the maxillary incisors and canines, incisal edge, and labial surface of the mandibular incisors and canines, and fossa area of the premolars and first molars (as illustrated in Figure 2).

### 2.4. Subject Teeth

The subject teeth were the maxillary (Mx) and mandibular (Mn) incisors (I), canines (C), second premolars (P), and second molars (M). The sensing point for the maxillary incisors was set on the labial surface at the middle of the right and left central incisors because the maxillary incisor becomes palatally inclined after the use of the MAD. In contrast, the sensing point for the mandibular incisors was set on the lingual surface, considering the force vector of the MAD. The buccal surfaces were chosen as the sensing points for other teeth.

### 2.5. Statistical Analysis

Each variable was measured 30 times by a single examiner (S–Y.L.). Descriptive variables were described as means and standard deviations. The Shapiro–Wilk test could not confirm the normality of data distribution in some variables. The comparison between models in each tooth or the comparison between tooth positions in each model were performed using the Kruskal–Wallis test in maxillary and mandibular dentition, separately. Post hoc comparison was performed using the Bonferroni test. Regression analysis was done to interpret the interaction between models and tooth position using generalized linear model (GLM).

Statistical significance was set at *p* < 0.05, and the hypothesis analysis was two-sided for all tests. Analyses were performed using SAS 9.4 (SAS Institute Inc., Cary, NC, USA).

## 3. Results

### 3.1. Overall Force Distribution According to the Tooth Site in Each Model

**Figure 3:**
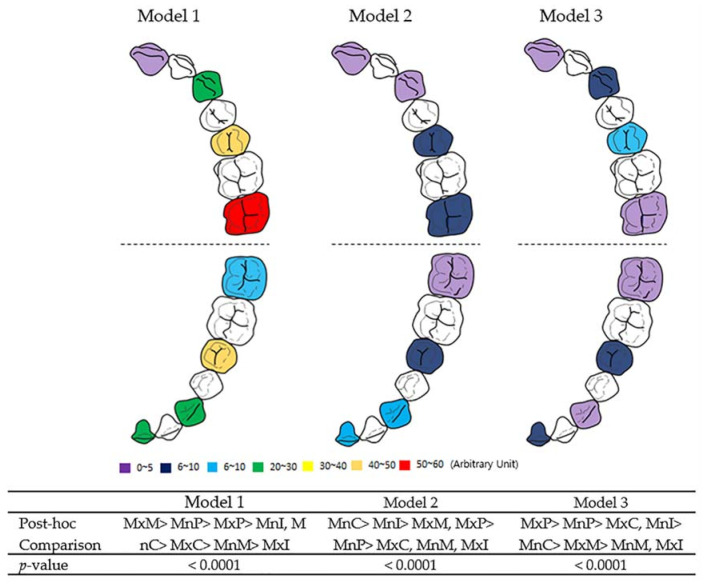
Overall force distribution according to tooth site in each model. Model 1, single-layer MAD; model 2, double-layer MAD; model 3, core-reinforced multilayered MAD; I, incisor; C, canine; P, second premolar; M, second molar; Mx, maxillary; Mn, mandibular. One-way ANOVA test and posthoc Games–Howell test were performed. The data regarding force values are described in Table 1 and Table 2.

Comparing the forces applied to the maxillary and mandibular teeth in each model, there was a significant difference in the force distribution depending on the tooth position (as illustrated in Figure 3). In model 1, the measured force decreased in the order of the maxillary second molar, mandibular second premolar, maxillary second premolar, mandibular incisor and canine, maxillary canine, mandibular second molar, and maxillary incisor (*p* < 0.0001). In model 2, the force value decreased in the order of the mandibular canine, mandibular incisor, maxillary second molar and second premolar, mandibular second premolar, maxillary canine, mandibular second molar, and maxillary incisor (*p* < 0.0001). For model 3, maxillary and mandibular second premolar showed the highest force values, followed by the maxillary canine, mandibular incisor, mandibular canine, maxillary and mandibular second molar, and maxillary incisor (*p* < 0.0001).

Regardless of the type of model, the mandibular incisors showed higher force values than the maxillary incisors and the force value of the maxillary second molars was higher than that of the mandibular second molars. In all three models, the maxillary incisors and mandibular second molars showed the lowest force values.

### 3.2. Force Distribution in the Maxillary Dentition

#### 3.2.1. Comparison between Models

**Figure 4:**
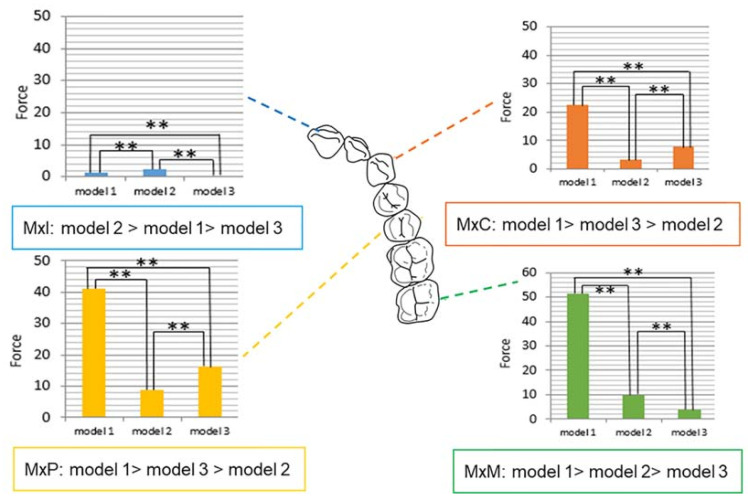
Force distribution in maxilla according to tooth position and models. Model 1, single-layer MAD; model 2, double-layer MAD; model 3, core-reinforced multilayered MAD; Mx, maxillary; I, incisor; C, canine; P, second premolar; M, second molar; Statistically significant (** *p* < 0.0001).

For the maxillary central incisors, the greatest force was observed in model 2, followed by that in model 1 and model 3 (*p* < 0.0001, as illustrated in Table 1). The maxillary canine and premolar areas had a similar force distribution pattern between the models, and model 1 showed the greatest force, while model 2 showed the lowest (model 1 > model 3 > model 2, *p* < 0.0001, as illustrated in Table 1). In the second molar area, model 1 had the highest force value, followed by model 2 and model 3 (*p* < 0.0001, Table 1) (as illustrated in Figure 4).

There were two variables in this study: tooth position and device material (models). When we compared model 3 with the other models in terms of compensation for the tooth position, only model 2 showed a significant difference (*p* = 0.0003, as illustrated in Table 3).

#### 3.2.2. Comparison between Tooth Positions

Regardless of the model, the maxillary incisors showed the lowest force among the different tooth types. In model 1, the measured force decreased in the following order: molar, premolar, canine, and incisor (*p* < 0.0001, Table 1). Model 2 showed a tendency similar to that of model 1; however, there was no significant difference between the molar and premolar areas (*p* < 0.0001, as illustrated in Table 1). In model 3, the premolars showed higher force values than those of canines, followed by the molars, and the incisor area had the lowest value (*p* < 0.0001, as illustrated in Table 1).

The maxillary canines, premolars, and molars revealed significant differences compared to the maxillary incisors when compensating for MAD materials (all, *p* < 0.0001, as illustrated in Table 3).

### 3.3. Force Distribution in the Mandibular Dentition

#### 3.3.1. Comparison between Models

**Figure 5:**
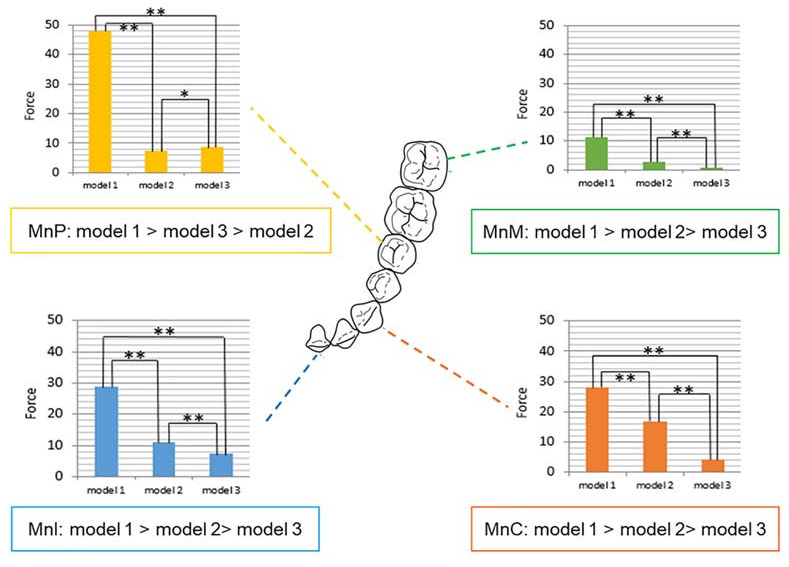
Force distribution of mandible according to tooth position and models. Model 1, single-layer MAD; model 2, double-layer MAD; model 3, core-reinforced multilayered MAD; Mn, mandibular; I, incisor; C, canine; P, second premolar; M, second molar; Statistically significant difference (* *p* < 0.001; ** *p* < 0.0001).

Except for the premolar area, all mandibular teeth showed the same tendency of force distribution. Model 3 showed the lowest force value in the mandibular incisors, canines, and molars, whereas the highest force values were observed in model 1 (model 1 > model 2 > model 3, all, *p* < 0.0001, as illustrated in Table 2). The force values of model 2 were lower than those of the other models only in the premolar area (model 1 > model 3 > model 2, *p* < 0.0001, Table 2) (as illustrated in Figure 5).

When the variables for tooth position were corrected, model 3 showed a significant difference from models 1 and 2, respectively (both, *p* < 0.0001, as illustrated in Table 4).

#### 3.3.2. Comparison between Tooth Positions

In all models, the mandibular second molars showed a significantly lower force than that shown by other teeth. In models 1 and 3, the force delivered to the tooth surface decreased in the following order: premolar, incisor, canine, and molar (model 1, *p* < 0.0001; model 3, *p* < 0.0001, as illustrated in Table 2). Model 2 showed a different pattern compared to other models, with the force decreasing in the following order: canine, incisor, premolar, and molar (*p* < 0.0001, as illustrated in Table 2). Similar to the maxillary dentition, the mandibular canines, premolars, and molars revealed significant differences compared to that of the mandibular incisors when compensating for MAD materials (*p* < 0.0001 for the canine and molar, and *p* < 0.05 for the premolar, as illustrated in Table 4).

## 4. Discussion

The efficacy of MADs depends on several factors including the severity of OSA, design of MADs, and degree of mandibular protrusion [27]. Regarding the design of MADs, most studies focused on the features of mono-bloc versus duo-bloc or custom-made versus prefabricated. There is no definite consensus on which design is the most effective, and a wide variety of vertical dimension of MADs were reported ranging from 1–14 mm [27,28]. The amount of bite opening should be minimized in the aspect of patient tolerance and treatment efficiency. Mayoral et al. [28] evaluated 4 levels of vertical opening from 2 mm to 11 mm and showed that increase of vertical dimension caused retrusive position of the mandible. Similarly, a recent meta-analysis showed that the mono-bloc type showed a greater reduction in apnea/hypopnea index (AHI), and an increase in oxygen saturation compared to the effects of the duo-bloc type [29]. Mono-bloc MADs generally allow lower vertical increase and less clockwise rotation of the mandible than duo-bloc MADs; therefore, we selected mono-block designs with similar thicknesses in this experiment. In terms of fabrication methods, the appropriate fit of MADs is an important prerequisite for treatment success. Traditionally, MADs were individually fabricated by a dental technician from plaster casts and construction bites obtained by the dentist. Recently, various prefabricated thermoplastic MADs appeared on the market, and these boil and bite devices are likely not to fit intimately with teeth. They showed more compliance failure mainly because of insufficient overnight retention and turned out to be less effective than custom-made devices [30].

Little is known about the impact of the design of MADs on the side effects. Pépin et al. [31] reported that heat-molded prefabricated MADs showed early adverse effects, such as dental pain, temporomandibular pain, and muscular pain after two months of use. However, no study was conducted on the critical long-term side effects of dental changes. We first introduced multiple layers into the MADs and compared them with existing materials. Combining materials with different physical properties will maximize the advantages of each material.

Most retrospective studies on the dental side effects of MADs focused mainly on the changes in the inclination of incisors, overjet, overbite, and width of the arch [9,12,32,33]. The anterior teeth are particularly susceptible to side effects because the root surface of the anterior teeth is the smallest of the whole dentition. In addition, the mandible moves antero-posteriorly when a MAD is applied, so the restorative force is directly transmitted to the anterior teeth. However, recent FEM studies [23,34] showed that load concentration induced by MADs was greater on the posterior teeth than on the anteriors; therefore, it is necessary to evaluate force distribution on the overall dentition as well as on the anterior teeth.

The maxillary incisors showed the least force in the maxillary dentition throughout the models, whereas in the case of the mandibular dentition, the molars showed the least force regardless of the model. This was the main difference between maxillary and mandibular dentitions. The other teeth in the upper and lower dentition showed inconsistent results depending on the device type. When wearing a MAD, the mandibular masticatory muscles pull the protruded mandible back [9,17], and this force is then directly transmitted to the mandibular incisors. The mandibular molars are aligned in the direction of the mandible move back and therefore can resist this force. However, the anterior teeth may be exposed to significant forces because they are aligned perpendicular to the direction of the restorative force of the mandible. This may explain why the molars showed lower force values than the incisors in the mandibular dentition. On the other hand, the situation was different in the maxillary dentition. Unlike the mandible, the force value on the maxillary molars was higher than that on the maxillary incisors. The reason for this is not clear; however, the maxilla is fixed and attached to the cranial base, so it is presumed that the force from the MADs is transferred indirectly to the maxillary dentition.

Our results also highlighted that attention should be paid to the transverse dimension of the posterior teeth during the long-term use of MADs, as well as the anteroposterior movements of the incisors. Previously, Lee et al. [23] showed the highest force value in the mandibular second molar; therefore, it is likely to be inclined lingually. In contrast, in our study, the mandibular second molar indicated had the lowest force value. If the mandibular second molar is inclined lingually, the mandibular intermolar width would decrease. However, Pliska et al. [16] reported significant increases in the mandibular intercanine and intermolar width as a consequence of the long-term use of MADs. In addition, the use of functional appliance, which has similar mechanism of action with MADs, also showed that mandibular premolar and molar widths were increased [35,36].

Model 1 consists of a single-layer PETG MAD. PETG is a noncrystallizing amorphous copolymer of polyethylene terephthalate (PET) and has good mechanical properties, formability, optical qualities, fatigue resistance, and dimensional stability [37,38]. Therefore, PETG is widely used to make orthodontic clear retainers or clear aligners. PETG is a relatively hard material, and it is suitable for the maintenance of the orthodontic treatment results. However, when used in the fabrication of MADs, it transfers the mandibular restorative force to the dentition rather than absorbing the force. In our study, model 1 showed higher force values than the other models in almost every tooth position. According to Ahn et al.’s study [39] on the aging procedure of PETG in an intraoral environment, the ultimate tensile strength and elastic modulus of PETG increased after six months of intraoral use. Because MADs are usually worn on a long-term basis, MADs made of PETG can undergo changes in their physical characteristics, which can make a difference regarding the force distribution on the dentition [40].

Model 2 was a double-layered MAD consisting of an outer PETG and inner TPU layer. TPU is one of the most versatile engineering thermoplastics with elastomeric properties [41]. TPU has several favorable properties, including excellent physical properties, chemical resistance, abrasion resistance, adhesion characteristics, and ease of processing [42,43]. Model 2 showed lesser force distribution than model 1 in all tooth positions except for the maxillary incisors, which may be because TPU is an elastic material, so it is more likely to deform and attenuate the applied forces [44]. However, delamination between the layers is frequent during the long-term use of double-layered MADs. Therefore, care must be taken in the clinical setting.

Model 3, which consisted of a core-reinforced multilayered MAD, an inner most reinforced resin core surrounded by a soft TPU and a hard PETG layer, had the lowest force values in the maxillary incisor and molar area. In the case of the mandibular dentition, model 3 showed the lowest force in all tooth positions except for the premolars. Because the only difference between models 2 and 3 was the presence of a resin core, the resin core is thought to have a critical role in reducing the forces applied by the device. It was selectively located at the incisal edge and palatal surface of the maxillary incisors, incisal edge and labial surface of the mandibular incisors, and fossa area of the posterior segment considering the vector of tooth movement when wearing a MAD. In addition, core integration contributes to the dimensional stability. The lateral wall of the appliance is well maintained without distortion or expansion, and thus, the arch width can be stabilized [45]. This may be the reason for the low force distribution of model 3 in our study.

The multilayered devices are inevitably thicker than the monolayer devices, and the vertical dimensions may influence the force distribution. Therefore, we tried to control the thickness and vertical dimensions of the three MADs. When designing the location of the resin core in the molar area, the fossa area was selected, and the thickness and vertical dimensions were similar to those of models 1 and 2. Considering that the interocclusal space at the physiological rest position is 2–4 mm from tooth contact, a 4 mm thickness is acceptable for most patients [46,47]. The total thickness of our MADs was 4–4.5 mm, which is assumed to be within the physiological range.

One shortcoming of our study was the lack of simulation of the periodontal ligament (PDL). The PDL has viscoelastic properties, and this causes a force decay under in vivo conditions [48]. In addition, thermoplastic polymers are highly viscoelastic materials, and temperature and humidity have significant effects on their mechanical properties [49]. Therefore, the mechanical characteristics may differ between intraoral and extraoral environments at room temperature. Since the clinical importance of multilayered MADs was confirmed, future studies are necessary to modify the core design depending on various malocclusion types. For example, in patients with deep bite, the core covering the incisal edge could be eliminated.

## 5. Conclusions

MADs exerted different force according to the tooth site, therefore it is necessary to understand overall force distribution on both entire arches.

The core-reinforced multilayered MADs reduced the force delivered to the dentition more effectively than conventional single-or double-layer devices, which would contribute to reducing long-term dental side effects of MADs.

## Figures and Tables

**Table 1:** Force distribution on maxillary dentition according to tooth site and MADs.

	Site	Incisors		Canines		Premolars		Molars		Comparison between Site	
Model		Mean	SD	Mean	SD	Mean	SD	Mean	SD	*p*-Value ^†^	
model 1		1.33	0.76	22.63	3.94	41.20	1.63	51.53	5.24	<0.0001	M > P > C > I
model 2		2.33	0.76	3.40	1.48	8.70	1.53	9.80	1.65	<0.0001	M, P > C > I
model 3		0.27	0.45	7.73	1.34	16.43	1.28	4.00	0.74	<0.0001	P > C > M > I
Comparison between models *p*-value ^†^		2 > 1 > 3		1 > 3 > 2		1 > 3 > 2		1 > 2 > 3			
		<0.0001		<0.0001		<0.0001		<0.0001			

Model 1, single-layer MAD; model 2, double-layer MAD; model 3, core-reinforced multilayer MAD; ^†^, Kruskal–Wallis test and post hoc Bonferroni test were performed; *p* < 0.05, significant difference. I, incisor; C, canine; P, premolar; M, molar.

**Table 2:** Force distribution on mandibular dentition according to tooth site and MADs.

	Site	Incisors		Canines		Premolars		Molars		between Site	
Model		Mean	SD	Mean	SD	Mean	SD	Mean	SD	*p*-Value ^†^	
Model 1		28.27	5.43	28.17	2.04	47.90	2.32	11.23	2.05	<0.0001	P > I, C > M
Model 2		11.07	1.55	16.93	1.82	7.53	1.07	2.70	0.92	<0.0001	C > I > P > M
Model 3		7.30	1.06	4.07	0.91	8.60	1.04	0.70	0.60	<0.0001	P > I > C > M
between models *p*-value ^†^		1 > 2 > 3		1 > 2 > 3		1 > 3> 2		1 >2 > 3			
		<0.0001		<0.0001		<0.001		<0.0001			

Model 1, single-layer MAD; model 2, double-layer MAD; model 3, core-reinforced multilayer MAD; ^†^, Kruskal–Wallis test and post hoc Bonferroni test were performed; *p* < 0.05, significantly different. I, incisor; C, canine; P, premolar; M, molar.

**Table 3:** The generalized linear model for model, tooth position, and interaction term between model and tooth position in the maxillary dentition.

Variable		Multivariable Generalized Linear Model (Dep = Value) in Mx			
		Estimate	95% CI		*p*-Value
model	1	1.07	−0.05	2.19	0.0617
	2	2.07	0.95	3.19	0.0003 **
	3	0.00	.	.	.
number	1 (I)	0.00	.	.	.
	3 (C)	7.47	6.35	8.59	<0.0001 ***
	5 (P)	16.17	15.05	17.29	<0.0001 ***
	7 (M)	3.73	2.61	4.85	<0.0001 ***
model (1) × number (1)		0.00	.	.	.
model (1) × number (3)		13.83	12.25	15.42	<0.0001 ***
model (1) × number (5)		23.70	22.12	25.28	<0.0001 ***
model (1) × number (7)		46.47	44.88	48.05	<0.0001 ***
model (2) × number (1)		0.00	.	.	.
model (2) × number (3)		−6.40	−7.98	−4.82	<0.0001 ***
model (2) × number (5)		−9.80	−11.38	−8.22	<0.0001 ***
model (2) × number (7)		3.73	2.15	5.32	<0.0001 ***
model (3) × number (1)		0.00	.	.	.
model (3) × number (3)		0.00	.	.	.
model (3) × number (5)		0.00	.	.	.
model (3) × number (7)		0.00	.	.	.

Mx, maxilla; I, incisor; C, canine; P, premolar; M, molar; statistically significant differences: ** *p* < 0.001, *** *p* < 0.0001.

**Table 4:** Generalized linear model for model, tooth position, and interaction term between model and tooth position in mandibular dentition.

Variable		Multivariable Generalized Linear Model (Dep = Value) in Mn			
		Estimate	95% CI		*p*-Value
Model	1	20.97	19.89	22.05	<0.0001 ***
	2	3.77	2.69	4.85	<0.0001 ***
	3	0.00	.	.	.
Number	1 (I)	0.00	.	.	.
	3 (C)	−3.23	−4.31	−2.15	<0.0001 ***
	5 (P)	1.30	0.22	2.38	0.0184 *
	7 (M)	−6.60	−7.68	−5.52	<0.0001 ***
model (1) × number (1)		0.00	.	.	.
model (1) × number (3)		3.13	1.61	4.66	<0.0001 ***
model (1) × number (5)		18.33	16.81	19.86	<0.0001 ***
model (1) × number (7)		−10.43	−11.96	−8.91	<0.0001 ***
model (2) × number (1)		0.00	.	.	.
model (2) × number (3)		9.10	7.57	10.63	<0.0001 ***
model (2) × number (5)		−4.83	−6.36	−3.31	<0.0001 ***
model (2) × number (7)		−1.77	−3.29	−0.24	0.0235 *
model (3) × number (1)		0.00	.	.	.
model (3) × number (3)		0.00	.	.	.
model (3) × number (5)		0.00	.	.	.
model (3) × number (7)		0.00	.	.	.

Mx, maxilla; I, incisor; C, canine; P, premolar; M, molar; statistically significant differences: * *p* < 0.05, *** *p* < 0.0001.

## Data Availability

Data sharing not applicable.
